# Diphyllobothriasis Caused by the Sanada Tapeworm: A Case Report

**DOI:** 10.7759/cureus.61147

**Published:** 2024-05-27

**Authors:** Masazumi Miyahara, Kyoko Osaki

**Affiliations:** 1 Department of Pediatrics, Okanami General Hospital, Iga, JPN

**Keywords:** anthelmintic, food parasitology, praziquantel, parasite, diphyllobothrium nihonkaiense, diphyllobothriasis, children

## Abstract

The incidence of human diphyllobothriasis is expected to rise amidst the current global popularity of Japanese cuisine, such as sushi, which contains raw fish. We report a case of a 10-year-old boy with a diphyllobothriasis infection acquired via sushi consumption. The patient was otherwise healthy, exhibited no symptoms, and was successfully treated with a single dose of 10 mg/kg praziquantel. In Japan, this parasite is known as “Sanada-mushi” because it resembles a Sanada cord. Prompt recognition of this parasite by evoking the Sanada cord’s appearance may facilitate early diagnosis and treatment and increase public awareness to prevent diphyllobothriasis.

## Introduction

In recent times, parasites have remained a human health hazard worldwide. Human parasitic infections typically occur through the ingestion of contaminated food or water or from the invasion of larvae through exposed skin, with the parasites living in or on the host and causing illness by consuming nutrients or damaging tissues. Parasites can be divided into two main groups: protozoa and helminths. Helminths are multicellular, multisystem organisms, often with complex life cycles [1]. Diphyllobothriasis, which is one of the helminth infections, is the most common fish-borne cestodiasis worldwide and uses two intermediate hosts to complete its life cycle. The first intermediate host is probably brackish zooplanktonic copepods, which are consumed by the second intermediate host, Pacific salmonids, namely cherry salmon, chum salmon, and pink salmon. In the second intermediate host, procercoids develop into plerocercoids, the larval form needed to infect the definitive host (e.g., humans, dogs, bears, and fish-eating birds) [2,3]. In particular, *Dibothriocephalus nihonkaiense* infection is reported occasionally in Japan due to the widespread consumption of raw or undercooked Pacific salmon, which possibly harbors the plerocercoid of this tapeworm. Although the incidence of diphyllobothriasis in Japan is estimated at approximately 40 to 60 cases per year, its incidence is probably much higher, and practically, the incidence has been estimated at 100 to 200 cases per year [2,4,5]. While diphyllobothriasis is generally asymptomatic or induces relatively mild gastrointestinal symptoms, such as diarrhea and pain, most patients experience mental distress, extreme defecation, and proglottid discharge, with a small number of patients developing severe complications such as anemia or inflammation of the bile ducts or appendix [6]. With growing globalization and the rapid increase in tourism in Japan, the consumption of sushi (pieces of raw fish with rice and other ingredients) or sashimi (slices of raw fish), which are typical Japanese dishes, has also increased. Moreover, infections can also occur in other countries and regions with other traditional cuisines that involve the consumption of raw fish, such as smoked, pickled fish in parts of Europe, smoked fish in North America, and ceviche (fish and spices marinated in lime juice) in South America. Some of these other countries have not experienced parasitic outbreaks before, but in recent years, infections have been occurring through imported raw fish [7,8]. Consequently, the number of individuals afflicted with diphyllobothriasis is anticipated to increase, highlighting the necessity of raising awareness about this parasitic infection. Evoking the appearance of the Sanada cord, which resembles the *Diphyllobothrium* species known in Japan as “Sanada-mushi,” may enable prompt recognition of this tapeworm by the general public and facilitate quick diagnosis, treatment, and prevention. Herein, we report a case of a 10-year-old boy with diphyllobothriasis associated with sushi consumption and introduce the term “Sanada cord” to facilitate an associative diagnosis. This is the first case to link the Sanada cord with tapeworm, which is a unique contribution considering its Japanese origin.

## Case presentation

**Figure 1:**
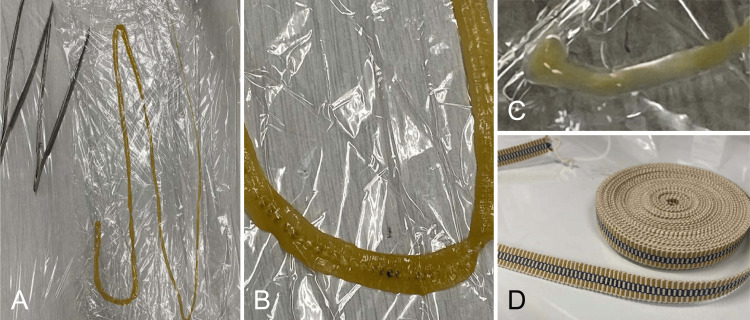
Sanada tapeworm expelled from the patient and Sanada cord A: The overall image of the extracted tapeworm. B: The magnified image of the tapeworm. C: The head of the excreted tapeworm. D: Sanada cord

A previously healthy 10-year-old boy was referred to our pediatric department after he found a string-like structure protruding from his anus. His mother pulled the string-like structure and tore off an approximately 1.5-m-long portion (Figures 1A, 1B). The patient had consumed raw salmon habitually, particularly sushi purchased from supermarkets near his residence once to twice a week, and details of the other foods consumed, which were obtained during the clinical investigation, were sparsely recollected by the patient and his family. The patient’s body temperature was 36.4 °C, and he exhibited no symptoms. A physical examination showed no abnormalities, including no signs of anemia. Blood tests or imaging studies were not performed. After examining the strobila of the severed portion of the string-like structure, the patient was diagnosed with diphyllobothriasis. A single praziquantel dose of 10 mg/kg was prescribed, resulting in the anal excretion of the tapeworm’s head (Figure 1C) and no further recurrence. Furthermore, at the initiation of treatment, we advised the patient to avoid consuming raw salmon in the future.

## Discussion

Diphyllobothriasis is a parasitic infection caused by broad or fish tapeworms. To date, six species (*Dibothriocephalus latus, Dibothriocephalus dendriticus, Dibothriocephalus nihonkaiense, Adenocephalus pacificus, Diphyllobothrium balaenopterae, and Diphyllobothrium stemmacephalum*) have been confirmed as true human parasites [9]. Due to the similarity in morphology and the effects of treatment or fixing agents on the morphology, identifying distinct *Diphyllobothrium* species requires molecular techniques. As the treatment for these species is the same, an exact species diagnosis is clinically unnecessary. Among these six species, the *Dibothriocephalus nihonkaiense* infection, closely associated with consuming raw Pacific salmon, is the most frequently occurring foodborne parasitic infection in Japan [4]. Because of the epidemiology and morphology, our case is considered to be a *Dibothriocephalus nihonkaiense* infection.

Paleoparasitologic studies have revealed that diphyllobothriasis has been occurring in Japan for approximately 1,000 years [10]. In Japan, the tapeworm *Dibothriocephalus nihonkaiense* is commonly known as “Sanada-mushi” due to its resemblance to the flat, woven Sanada cord (Figure 1D), which was named after the Sanada clan of samurai warriors who fought against Tokugawa Ieyasu, the founder of the Edo Shogunate, during the Warring States phase of Japanese history. A Sanada cord (“Sanada-himo” in Japanese) is a flat cord woven from cotton or silk. It is used to secure tea utensils, swords, armor, and obi ties and is also used for other purposes, such as tying bundles. While well-known in contemporary Japan, both the Sanada clan and Sanada cords may be unfamiliar to many individuals. Hence, Sanada cords may not be commonly associated with a parasitic worm.

Diphyllobothriasis affects an estimated 10-20 million people worldwide in modern times [6]. This high number is partly attributed to the lack of awareness of the risks associated with consuming raw fish. Diphyllobothriasis is usually asymptomatic but anemia due to vitamin B12 deficiency (especially in *D. latum* infection); abdominal symptoms such as pain, discomfort, constipation, and diarrhea; weight loss; fatigue; myalgia; headaches; and dizziness have been reported [6,11-13]. Moreover, the persistent excretion of worms from the anus may be substantially emotionally burdensome for patients and their families. Adult tapeworms of this species reside in the small intestine of their vertebrate host and are folded in loops. Attachment to the intestinal wall usually takes place at the level of the ileum and less commonly in the jejunum or other levels. If left untreated, adult parasites may survive for decades in the human host [2,6]. Preventing this parasitic disease is thus important. In our case, the consideration of sushi as the source of infection, the asymptomatic nature of the patient, and the detection of the infection due to the expulsion of the parasite from the anus were similar to the presentation in other typical cases of diphyllobothriasis. Although atypical cases, manifesting aberrant migration of proglottids or massive infection, can cause cholecystitis, cholangitis, intestinal obstruction, or vitamin B12 deficiency [11,14], such serious complications did not occur in our case, which could be partly attributed to the high possibility of *Dibothriocephalus nihonkaiense* infection, early diagnosis and treatment, and good response to treatment.

The current treatment of choice is praziquantel [15]. Praziquantel is ingested but not metabolized by the parasite, interfering with calcium metabolism and causing flaccid paralysis. The Centers for Disease Control and Prevention recommend oral administration of a single dose (5-10 mg/kg) of praziquantel in children and adults [16]. The patient’s stool should be examined for ova and parasites one month after treatment to ensure the eradication of the parasite. In this case, the parasite was successfully eradicated using this treatment. The best prophylaxis is to avoid the consumption of raw, smoked, or pickled fish. Fish should be well cooked; alternatively, deep freezing is an effective way of killing the parasites. The methods recommended by the US Food and Drug Administration are that fish should be frozen and stored at an ambient temperature of -20 ℃ or below for seven days, frozen at an ambient temperature of -35 ℃ or below until solid and stored at an ambient temperature of -35 ℃ or below for 15 hours, or frozen at an ambient temperature of -35 ℃ or below until solid and stored at an ambient temperature of -20 ℃ or below for 24 hours to prevent infection. However, it should be noted that these conditions may not be suitable for freezing particularly large fish (e.g., thicker than 6 inches) [17]. On the other hand, this practice has not been implemented for the fishborne parasites *Diphyllobothrium* species in Japan [2]. Globalization has further complicated the epidemiology of parasitic infections. In Japan, diphyllobothriasis endemic areas were previously limited to Hokkaido Prefecture, Tohoku region, and the coastal region on the Sea of Japan, where salmon are caught and consumed locally; however, diphyllobothriasis cases have been reported recently across Japan, and it occurred most often in Tokyo and Saitama, the populous cities with the rapid advancement of food transportation systems and techniques to retain freshness [2]. In this case, the patient resided in a less populous region without nearby sea access and was likely infected via the consumption of sushi topped with raw salmon transported from coastal areas.

To eradicate these parasitic diseases, the risk of parasitic infection associated with the consumption of raw fish must be adequately recognized by healthcare professionals and the public including consumers, food producers, restaurant owners, and visitors. Moreover, it is important to ensure that the aforementioned freezing processes are thoroughly applied during fish storage, processing, and preparation. Once the infection has been established, a high index of suspicion, thorough patient history, and the asking of occupational history, travel history, and eating habits can lead to early diagnosis. Similarly important is the swift identification of asymptomatic tapeworm carriers, for which the following methods could be effective: conducting regular health screenings and monitoring, including stool tests, particularly in regions or among people who frequently consume raw fish; encouraging individuals with a high risk of the infection to report even minor symptoms to enable early diagnosis; and developing highly sensitive rapid tests to identify infections. Particularly, raising widespread awareness about the association between eating raw salmon and parasitic infections among the general public is crucial. We suggest that associating this parasite with a specific name, such as “Sanada” tapeworm, is highly meaningful, and awareness may be accordingly disseminated.

## Conclusions

Human diphyllobothriasis may become a greater social concern soon because of the increasing global popularity of eating raw fish. Educating the general public to recognize the risk of the consumption of raw fish as well as appropriate food preparation is paramount for the prevention of this parasitic infection. The parasites strikingly resemble woven Sanada cords, which have been used in Japan since the Warring States period. Associating the parasite with the Sanada cord may be effective not only for early diagnosis and treatment but also in promoting awareness and prevention of diphyllobothriasis. Once the infection occurs, it is important to quickly screen the transmission route from the consumed food and detect infected individuals early, including asymptomatic ones, to ensure a favorable outcome, control transmission, and prevent future outbreaks.
